# Common Factors of Psychotherapy in Inpatients With Major Depressive Disorder: A Pilot Study

**DOI:** 10.3389/fpsyt.2019.00463

**Published:** 2019-07-02

**Authors:** Kathrin Woike, Eun-Jin Sim, Ferdinand Keller, Carlos Schönfeldt-Lecuona, Zrinka Sosic-Vasic, Markus Kiefer

**Affiliations:** ^1^Department of Psychiatry and Psychotherapy III, Ulm University, Ulm, Germany; ^2^Department of Child and Adolescent Psychiatry and Psychotherapy, Ulm University, Ulm, Germany

**Keywords:** major depressive disorder (MDD), psychotherapy, cognitive–behavioral psychotherapy, common factors, treatment outcome evaluation

## Abstract

**Background:** Psychotherapeutic interventions share common factors, which might contribute to treatment success independent of the type of psychotherapy. Previous research on common factors of psychotherapy was mostly conducted in outpatients and covered the development of common factors throughout a therapy over months or years. However, the role of common factors for the psychotherapeutic treatment success in inpatients during their hospital stay has not been addressed so far. The present research therefore aimed to explore changes of the common factors within a short-term stay at the psychiatric hospital for inpatients with major depressive disorder (MDD) and their relation to treatment outcome.

**Method:** We developed a standardized manualized individual cognitive–behavioral psychotherapy (SMiCBT) for depression. The SMiCBT treatment lasted 4 weeks with eight therapy sessions. Following each treatment session, patients and therapists separately completed the questionnaire of “Stundenbogen für die Allgemeine und Differentielle Einzel-Psychotherapie” (STEP) to assess common factors from the perspective of the patient and the therapist. Severity of depression was also measured by the German version of the “Beck Depression Inventory” (BDI-II) before and after the treatment (SMiCBT). We conducted multilevel analysis for the longitudinal data for each scale of the STEP.

**Results:** We found an improvement in the severity of depressive symptoms across the treatment period according to BDI-II scores. Regarding the STEP scales, motivational clarification and problem-solving scores increased over the treatment period for both patient and therapist perspectives. This was not the case for the scale therapeutic relationship. Furthermore, baseline levels of motivational clarification and problem solving were related to the treatment response.

**Limitations:** The results have to be interpreted with care because of the small sample with MDD and the lack of a control group for comparison of treatment outcome.

**Conclusion:** Our data demonstrate that common factors improve within a short-term psychotherapy in inpatients with MDD. Most importantly, our research highlights the distinguished role of motivational clarification and problem solving for the improvement of depressive symptoms during short-term psychotherapy in inpatient settings.

## Introduction

Major depressive disorder (MDD) is one of the most common and examined psychiatric disorder (1). Many studies focused on understanding the origin and maintenance of this disorder (2). Psychotherapy is an efficient option for treating MDD, in particular in combination with psychopharmacological therapy (3). Different schools and techniques of psychotherapy result in similar treatment outcome for patients, known as the “Dodo bird verdict” (4). Hence, in addition to the specific techniques used in the therapeutic process, there have to be shared characteristics of the therapy, which might lead to an effective improvement in the patient’s condition. For this reason, various studies examined common factors of psychotherapy, which contribute to treatment success in every type of psychotherapy (5). Common factors are distinguished from specific or unique factors that belong to a special form of psychotherapy such as cognitive reframing in cognitive–behavioral therapy (6, 7). Whereas one of the most common classification of those factors is the generic model of psychotherapy (8), the common factors of Grawe (9) are well known and extensively examined in the German-speaking countries (10). The common factors of Grawe include 1) therapeutic relationship, 2) activation of resources, 3) actualization of the patient’s problems, 4) motivational clarification, and 5) (active help for) problem solving. According to Grawe (9), these five common factors have per se an effect on the outcome of the therapeutic process independent of the specific technique (11, 12). Several studies suggest that therapeutic relationship should be given relatively more attention than other common factors (13). For instance, a review study (14) showed that the most consensual commonalities among these common factors were the development of a therapeutic alliance, i.e., a well-aligned working relationship between the patient and therapist (15). In general, among all common factors, therapeutic alliance has been proven previously to have the most robust predictive value on therapeutic outcome (16–18). In a recent meta-analysis by Flückiger and colleagues (19), robustness of the positive relation between the alliance and outcome has been confirmed. However, this association has mainly been investigated based on data of the therapeutic alliance at the beginning of a psychotherapeutic process and not during the course of the therapy. In contrast, very little knowledge about the predictive value of common factors during the time course of a therapy is available, even though it might reveal additional valuable information. For example, outcome expectancy was found to change during the time course of therapy within patients as well as therapists, and was associated with therapy outcome (20). Regarding common factors, there is an increasing number of studies indicating that they might change over the course of a psychotherapy. Solomonov and colleagues (14) found fluctuations in the relation between the applications of common factor techniques by therapists and the development of therapeutic alliance, as reported by the patients. In addition, in the psychoanalytic literature, the observation of a phenomenon called “rupture and repair” is well known and acknowledged and refers to a pattern of increases and decreases in the therapeutic alliance, especially the working alliance (21–25) over the course of treatment. With respect to cognitive–behavioral therapy, there are few studies indicating a linear time course of the therapeutic alliance during the cognitive therapy (26). For example, Hoffart et al. (27) found therapist-rated alliance during the so-called residential cognitive therapy to increase with time, whereas patients-based alliance rating followed a U-shaped (quadratic) pattern over the course of treatment (10 weeks). Furthermore, weekly measured fluctuations in common factors were associated with subsequent fluctuations in outcomes. Thus, in summary, there is some, even though at the current stage rather sparse, evidence of a specific time course of common factors and its possible association with therapy outcome. However, in these previous studies, only working alliance was assessed. In addition, most studies on common factors were based on data from outpatients (28) or therapists frequently evaluated various therapeutic methods (15) and often pictured the development of common factors over months or years (29). In contrast, the psychotherapeutic treatment of depressed inpatients in psychiatric inpatient settings frequently typically includes only a short-term treatment of some weeks, because the hospital stay of patients is temporally restricted (no more than 3 to 5 weeks) (30). It is well known that techniques of psychotherapy are effective in the treatment of MDD, even if they are used in a short period of time with interventions between 4 and 10 sessions (31, 32). Nevertheless, it must remain open, whether the common factors of psychotherapy unfold their effect on patients’ clinical improvement in the setting of a short-term psychotherapy for MDD in a psychiatric hospital. Moreover, although several studies (12, 13, 18, 33) investigated psychotherapy from a common factors perspective, studies relating individual differences in common factors to treatment response are scarce. Altogether, there is still a considerable debate about the differential importance of common factors for the success of psychotherapy in different treatment settings.

The present pilot study therefore aims to explore the time course of common factors in the psychotherapeutic treatment of inpatients with MDD within a short stay of about 4 weeks in a psychiatric hospital and examines their relation to therapeutic outcome. Our observation period of 4 weeks conforms to the typical length of a hospital stay in MDD patients in Germany. For the purpose of our study, we developed a standardized manualized individual cognitive–behavioral psychotherapy (SMiCBT), including common cognitive–behavioral therapy techniques (34). We measured common factors according to Grawe using an established standardized questionnaire (Stundenbogen für die Allgemeine und Differentielle Einzel-Psychotherapie: STEP) (35). We choose to investigate common factors based on Grawe’s postulation, because they are related to the outcome of the therapeutic process independent of the specific psychotherapeutic intervention techniques (11). Grawe’s common factors are best displayed in the STEP questionnaire, which assesses the relevant factors motivational clarification, problem solving, and therapeutic relationship. The subscale structure of STEP has been validated by means of scale and factor analysis (36). Additionally, the STEP questionnaire is designed to evaluate an individual therapeutic session, which allows us to investigate changes in common factors across the entire treatment period. Treatment success was measured using the German version of the “Beck Depression Inventory” (BDI-II) (37, 38). We administered the BDI-II twice, in the first session at the beginning of the treatment (BDI-II at T0) and after the last session of SMiCBT at the end of the fourth week (BDI-II at T1).

We assume that the common factors “problem solving,” “motivational clarification,” and “therapeutic relationship” change during the course of the therapy for the following reasons: First, “problem solving,” as measured by STEP, should increase during the course of the therapy, because this is an essential goal of behavioral therapy to promote the patient’s self-management competences. Accordingly, with an increase of the patient’s capacity in “problem solving,” a positive adjustment in behavior and thus a reduction of depressive symptoms should be observable. Second, “motivational clarification,” the next subscale of STEP, should also increase during the course of the therapy, because, even though goals and motives are clarified at the beginning of each standard CBT, patient’s motivation to change unfavorable thoughts or behaviors should be enhanced from session to session, for example, during the course of several socratic dialogues between patient and therapist. Thirdly, “therapeutic relationship,” the third subscale of STEP, is also expected to show a positive development during the course of the therapeutic process, since mutual trust, but also working alliance should become more apparent, at least in cases where the therapy is continued and not interrupted. Lastly, we expect that the change of common factors over therapy sessions would be associated with improvements in depressive symptoms after treatment.

## Methods

### Participants

The study was conducted between November 2015 and July 2017 at the Department of Psychiatry and Psychotherapy III of the Ulm University. All participants were inpatients with MDD diagnosed according to *Diagnostic and Statistical Manual of Mental Disorders, Fourth Edition* (*DSM-IV*) criteria by an experienced psychiatrist and were treated in one of two open wards or in the day care ward. According to this screening procedure, the inpatients with a current or lifetime comorbid *DSM-IV* diagnosis of schizophrenia, other psychotic disorders, anxiety disorder, bipolar disorders, organic psychosis, current substance abuse, or dementia were excluded. Additional exclusion criteria were a medical or neurological illness of sufficient severity to interfere with the evaluations and interviews for our study. Finally, only inpatients with MDD as principal diagnosis participated in the present study. We applied this strict criterion for our study to exclude a potential confounding influence of comorbid psychiatric disorders.

Besides a treatment as usual such as occupational therapy, music therapy, sport therapy, and art therapy, the participants obtained an SMiCBT (see details on the section Study Design). The SMiCBT was conducted by clinical psychologists undergoing an intensive training in psychotherapy (psychotherapist in training, PiT). Seventeen PiTs were involved in the study. All PiTs were introduced to the SMiCBT and trained by a qualified psychotherapist before the beginning of the study, in order to standardize psychotherapeutic treatment and avoid inter-therapist differences. Each PiT treated at least one patient, maximum three patients, whereas no patient had more than one PiT during the therapeutic treatment. This study was carried out in accordance with the recommendations of the ethics committee of Ulm University (169/12-CL/Sta). The protocol was approved by the ethics committee of Ulm University. All subjects gave written informed consent in accordance with the Declaration of Helsinki. The patients participated voluntarily after providing written informed consent.

We recruited 32 inpatients. Data of seven patients who did not complete enough therapeutic sessions (less than six sessions) were excluded from data analysis. As a result, data analysis was conducted with a relatively small sample size of 25 participants (20 female). Most of the patients (92%) received antidepressants that were adjusted by clinical judgment based on the international clinical guidelines for the psychopharmacotherapy of depression (39). Seventy-six percent of the patients used three or more classes of the prescription drugs (for details, see **Table 1**).

**Table 1:** General information about the sample.

	Total sample	Group 0	Group 1	χ^2^/*t* test between Group 0 and 1
*N*	25	8	17	
Female (*n*)	20	7	13	χ^2^(1) = 0.41
Age (range)	39.2 (18–58)	36.0 (18–50)	40.7 (19–58)	*t*(23) = −0.83
Years of education	10.48	10.25	10.59	*t*(23) = −0.52
Art therapy (%) Music therapy (%) Sport therapy (%) Occupational therapy (%)	80 40 76 96	87,5 50 75 87.5	76,5 35,3 76,5 100	
No. of sessions, mean (SD)	7.6	7.3 (SD = 0.39)	7.8 (SD = 0.89)	*t*(23) = −1.76
Use of psychopharmacotherapy (%) SSRI (%) SNRI (%) Dopamine RI (%) Tricyclic antidepressants (%) Benzodiazepines (%) Antipsychotics/anticonvulsants (%)	92 64 28 4 44 28 8	100 75 25 0 50 12,5 12,5	88.2 58.8 29.4 5.9 41.2 35.3 29.4	χ^2^(1) = 1.02
Duration of current episode (months)	7.4	7.9	7.1	*t*(23) = 0.31
Previous treatment (yes)	64%	87,5%	52,9%	χ^2^(1) = 2.82
BDI-II score T0 Mean (SD)	32.2 (9.74)	29.0 (SD = 6.09)	33.7 (SD = 10.88)	*t*(23) = −1.13
BDI-II score T1 Mean (SD)	20.2 (11.42)	27.4 (SD = 8.26)	16.8 (SD = 11.31)	*t*(23) = 2.35*
Paired *t* test between BDI-II Scores T0 and T1	*t*(24) = 5.89**	*t*(7) = 1.28	*t*(16) = 8.28***	

Group 0 = improvement in BDI sum score ≤ 8 points, Group 1 = improvement in BDI sum score ≥ 9 points, number of sessions and use of psychopharmacotherapy during current hospital treatment. BDI-II, Beck Depression Inventory; SD, standard deviation.

*p < .05, **p < .001, ***p < .0001.

### Assessment

At the beginning of the SMiCBT, we confirmed patients’ diagnosis of MDD by using the standardized interview “Diagnostisches Kurz-Interview bei psychischen Störungen” (Mini-DIPS) (40) and a structured interview based on *DSM-IV*. We also collected sociodemographic data. According to the goal of our study, we measured the common factors of psychotherapy by the STEP questionnaire (41). This questionnaire provides one version answered by the patient and another version answered by the therapist. Each version includes 12 items, which record the perspective of patient and therapist on the therapeutic process of each therapy session on a seven-step rating scale. For each perspective, there are three subscales, which measure three common factors of psychotherapy: “motivational clarification” (K-scale), “active assistance in problem solving” (P-scale), and “therapeutic relationship” (B-scale). We used BDI-II (37, 38) to assess depressive symptoms in MDD. The BDI-II is a 21-item measure of depression symptoms and showed good internal consistency (α = .92) and test–retest reliability (*r* = .93) (37). The German version of the BDI-II (38) also demonstrated good reliability [internal consistency (Cronbach’s alpha ≥ 0.84) and retest reliability *r* ≥ 0.75] (42). Because the items of BDI-II referred to depressive symptoms experienced during the previous 2 weeks including today, i.e., current terms, participants completed the BDI-II only twice during the therapy treatment, in the first session (BDI-II at T0) and at the end of the fourth week (BDI-II at T1), and not after each therapeutic session.

### Study Design

Based on the average duration of hospital stay, we assessed the patients during a period of 4 weeks with standardized psychotherapy. After the screening procedure, we included patients only with MDD as the main diagnosis and without comorbid disorder. In the study period of 4 weeks, the patients received individual SMiCBT with two sessions of 50 min each week. After each session (except the first session that was intended as initial interview), therapists and patients separately assess the three common factors of psychotherapy by completing the STEP. Our therapy program (SMiCBT) included established cognitive–behavioral therapy techniques (34). In the first session, patient and therapist got to know each other and symptoms and problems of the patient were identified (initial interview). In the second session, psychoeducational content about the origin of depressive symptoms and their influence were explained based on the diathesis-stress model (psychoeducation). The third session dealt with the organization of individual pleasant or positive activities of the patient (development of activities). In the fourth session, the relation between cognition and emotion was analyzed by using the 5-split technique (documenting an activating event, the thoughts and emotions of the patient, and alternative thoughts with different emotional outcome for the same situation; cognitive reframing/reorganization). With reference to this technique, typical dysfunctional thoughts, attitudes, and beliefs were identified in session 5 (depressive mental bias). These dysfunctional cognitions were reappraised in more realistic or positive ones in session 6 and the patient learned about positive self-instruction (self-verbalization). In session 7 (handling of aversive emotions), patients were provided with helpful techniques for dealing with aversive emotions (like opposite acting). In session 8 (conclusion), the content of the therapy was summarized. The patients received homework after each session, and at the beginning of each session, there was time for answering questions. The participants completed the BDI-II at the first therapy session (T0) and after completion of their therapy (T1).

### Data Analysis

A multilevel analysis for the longitudinal data was performed for each subscale of the STEP. Following the approach in Hox (43), the basic model contains only an intercept term and variances at the repeated measures (level 1) and the subject level (level 2). This intercept-only model (null model) estimates whether the variance at the subject level is significant, i.e., whether the person-specific intercepts are heterogeneous. In the main model, the time variable (session number) was firstly added as a linear predictor. Since the time effect is considered fixed, the model predicts the course in each STEP subscale over the session numbers for all subjects simultaneously. Next, the predictor “change in depressive symptoms” was entered into the model. This predictor was defined by the difference of sum score of the reported BDI-II between the beginning of the study (T0) and after the last session of psychotherapy (T1). Based on BDI’s reliable change index (RCI) (44, 45), for this purpose, we split the whole group into two subgroups: group 1 (*n* = 17) showed a clinically relevant change in depression severity (improvement in BDI-II sum score ≥ 9 points). Group 0 (*n* = 8) did not show a clinically relevant improvement of depressive symptoms (improvement in BDI-II sum score ≤ 8 points). The final model consisted of the fixed effects (session number and improvement) and their interaction term, and the random effect intercept to account for the differences in subject level. Since the stepwise inclusion of the fixed effects revealed no relevant change to the results of the final model, only the results of the final model are reported (see **Table 3**). For the analyses of these models, we used the statistics software Proc Mixed from SAS 9.4 (46). Furthermore, we extended our statistical analysis to examine the association between the individual changes in common factors over therapy sessions and the individual change in depressive symptoms. As a measure for individual change in the common factors, we estimated the slope of the individual regression line as a function of session number of each patient, separately for each subscale of the STEP. Individual change in depressive symptoms was calculated as the difference of the BDI-II sum score before and after psychotherapeutic treatment (T0 − T1) for each patient. For the calculation of this analysis, we used the package stats of statistics software R (47).

## Results

### Participant Characteristics

Demographic and clinical features of participants are reported in **Table 1**. The average age of 25 participants was 39.2 years with a range between 18 and 58 years (SD = 13.09 years) (for details, see **Table 1**). Groups with clinically relevant improvement of depressive symptoms (group 1) and without such improvement (group 0) consisted of approximately 20% men and 80% women. This pronounced overrepresentation of female patients is typical for our psychiatric hospital. There was no significant difference between both improvement groups with respect to their number of attended treatment sessions (*t* = −1.76, *p* > .05). We also conducted a preliminary analysis to assess depressive symptoms of inpatients in both groups at the beginning of treatment and after treatment. Independent-samples *t*-tests demonstrated that there are only significantly different BDI-II sum scores between group 0 and group 1 after treatment (*t* = 2.35 *p* < .05, Cohen’s *d* = 1.07), but not before treatment (*t* = −1.13, *p* = .0.27, Cohen’s *d* = −0.54). A paired *t* test of BDI-II at T0 and BDI-II at T1 showed an improvement in severity of depressive symptoms after the treatment in total sample (*t* = 5.89, *p* < .0001, Cohen’s *d* = 1.37) as well as in group 1 (*t* = 8.28, *p* < .001, Cohen’s *d* = 1.52).

### Null Models

The intercept-only models (null models) for each subscale of the STEP showed that the subject-specific intercepts are significantly heterogeneous (see **Table 2**). The percentages of total variance that can be explained by the variance of interindividual differences of subjects in the intercepts varied between 50 and 75% on all subscales.

**Table 2:** Variances of the intercepts and tests of homogeneity.

	Intercept	*p*
STEPP-K	33.17	.001**
STEPP-P	14.46	.003**
STEPP-B	7.09	.001**
STEPT-K	25.87	.001**
STEPT-P	14.75	.004**
STEPT-B	4.80	.002**

*p < 0.05, **p < 0.01.

### Main Model

#### Development of Common Factors Across Treatment Period

**Figure 1:**
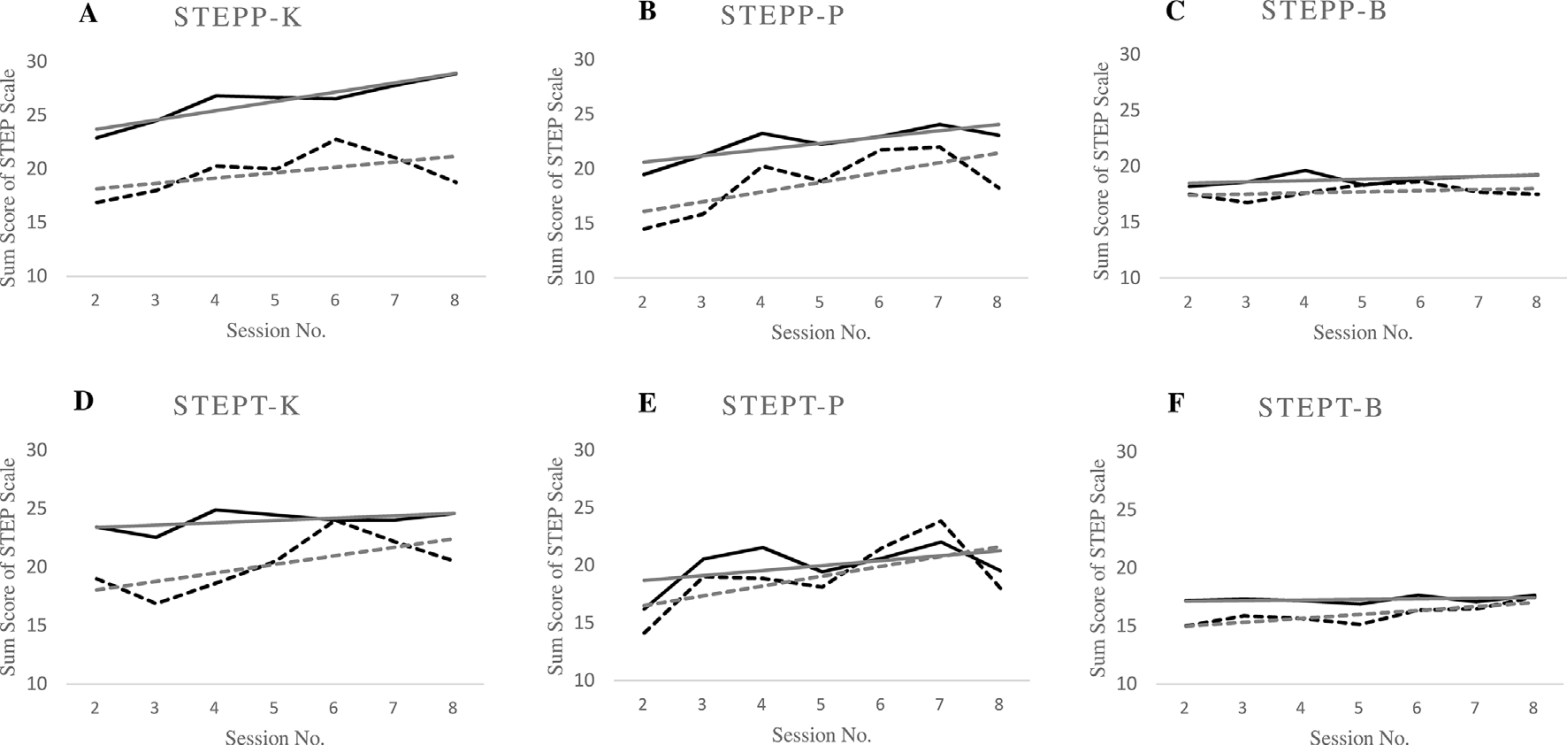
Changes in the sum scores of each “Stundenbogen für die Allgemeine und Differentielle Einzel-Psychotherapie” (STEP) scale in relation to therapeutic session number. Graphs **(A)** and **(B)** show main effects of change across time (number of therapeutic sessions) and Beck Depression Inventory (BDI) improvement. Graph **(D)** shows main effects of change across time (number of therapeutic sessions) and graph **(E)** shows main effect of BDI improvement. Graphs **(C)** and **(F)** show no significant effects of change across time (number of thrapeutic sessions) of patient and therapist between the word improvement. STEPP-K, patient, scale motivational clarifications; STEPP-P, patient, scale active assistance in problem solving; STEPP-B, patient, scale therapeutic relationship; STEPT-K, therapist, scale motivational clarification; STEPT-P, therapist, scale active assistance in problem solving; STEPT-B, therapist, scale therapeutic relationship, session number. Solid lines: group with improvement in BDI sum score ≥9 points; broken lines: group with improvement in BDI sum score ≤8 points; grey lines depict regression lines.

There was a significant main effect of change across time (number of therapeutic sessions) for the scale active assistance in problem solving (P scale) for evaluations of both patients and therapists (see **Table 3**; **Figures 1B, E**). Additionally, there was a significant main effect of change across time for the scale motivational clarification (K scale) only for the patients’ evaluation (see **Figures 1A, D**). For the scale therapeutic relationship (B scale), there were no significant effects of session number for evaluations of neither group (see **Figures 1C, F**).

**Table 3:** Tests of fixed effects change across time **(A)**, effects between groups with or without clinically relevant change in depressive symptoms **(B)**, and their interaction effect **(C)**.

	(A) Main effect: Change across time			(B) Main effect: improved vs. not improved			(C) Interaction effect: Change across time × improved vs. not improved	
Scale	*β*	*t*	*p*	Diff. between groups	*t*	*p*	*t*	*p*
STEPP-K	0.86	5.01**	<.001**	−6.26	−2.26*	.025*	0.18	.855
STEPP-P	0.53	3.06*	.003*	−6.34	−2.81*	.006*	1.92	.056
STEPP-B	0.07	0.95	.344	−1.65	−1.23	.222	0.94	.350
STEPT-K	0.25	1.53	.130	−6.55	−2.50*	.014*	1.80	.074
STEPT-P	0.41	2.20*	.029*	−3.47	−1.43	.156	1.49	.138
STEPT-B	0.02	0.27	.790	−2.29	−1.89	.061	0.95	.344

The number of degrees of freedom is df = 136 for all t tests. STEPP-K, patient, scale motivational clarification; STEPP-P, patient, scale active assistance in problem solving; STEPP-B, patient, scale therapeutic relationship; STEPT-K, therapist, scale motivational clarification; STEPT-P, therapist, scale active assistance in problem solving; STEPT-B, therapist, scale therapeutic relationship; *p < 0.05, **p < 0.01.

#### Association of Common Factors With Change in Depressive Symptoms

We compared the patient groups with and without clinically relevant changes in depressive symptoms with regard to the overall difference in the subscales of STEP over therapy sessions. We found a significant effect of patient group on the scales motivational clarification (K scale) for evaluations of patients as well as of therapists, and on the scale active assistance in problem solving (P scale) only for patients’ evaluations (see **Table 3B**). There were no significant differences on the scale therapeutic relationship (B scale) for patients’ and therapists’ evaluations.

Moreover, there was no significant interaction between therapy sessions and patient groups for neither scale (see **Table 3C**). There were only tendencies towards an interaction for the scale active assistance in problem solving (P-scale) of patients’ evaluations (*p* = 0.056) and for the scale motivational clarification (K-scale) for evaluations of therapists (*p* = 0.074).

### Correlations Between Slopes of the Individual Regression Functions as a Function of Session Number for STEP Subscales and Individual Changes in Depressive Symptoms

Spearman correlations between the slopes of the individual regression functions (as index of the development of the common factors across time) and changes in depressive symptoms (difference between BDI-II T0 and T1) varied between −.03 and .18 for the different STEP subscales and failed to reach significance (all *ps* > 0.16).

## Discussion

The aim of the present pilot study was to examine how common factors of psychotherapy change in inpatients with MDD, who received cognitive–behavioral psychotherapy within a study period of 4 weeks. In particular, we assessed whether these common factors were related to an improvement of depressive symptoms within the study period. Using an SMiCBT developed in-house for the treatment of depression, we measured three common factors with the STEP questionnaire: motivational clarification (K-scale), active assistance in problem solving (P-scale), and therapeutic relationship (B-scale). Severity of depression was assessed with the BDI-II before and after the SMiCBT. We expected that common factors would increase over the eight therapy sessions within the study period. We also expected an association between this increase in common factors and an improvement in depressive symptoms between the beginning and the end of the therapy treatment.

We found increases in common factors across the therapeutic sessions for the STEP scale active assistance in problem solving (P-scales) for both patients’ and therapists’ evaluations and for motivational clarification (K-scale) only for the patients’ evaluations. Recent meta-analyses have shown general large effects of providing problem-solving techniques to patients’ outcomes (48, 49), which, however, most likely first have to be trained and exerted by patients. In line with earlier work (9, 50), the present study demonstrated a development in these common factors across psychotherapy sessions. In contrast, we found no significant effect of session number for the scale therapeutic relationship (B-scale), neither for patients’ nor for therapists’ evaluations. Hence, in contrast to motivational clarification and active assistance in problem solving, the therapeutic relationship did not improve over the study period of 4 weeks in our therapeutic setting. This differential development of common factors during the course of the therapy might be due to their specific focus. Motivational clarification and active assistance in problem solving refer to the content of therapy, whereas therapeutic relationship is an interpersonal variable not directly targeted in our standardized therapy. The therapeutic relationship is often emphasized as an essential part of any psychotherapy to reach a positive therapy outcome (51). In research on therapeutic relationship, the most commonly discussed and evaluated concept regards “therapeutic alliance” [for instance, Ref. (19)]. Meta-analyses have shown moderate but consistent predictive values of therapeutic alliance to treatment outcome (18, 52, 53). Furthermore, evidence from other meta-analyses indicate relevance of therapeutic alliance to therapy outcome only for therapies that are relatively unstructured (54), whereas in more structured therapies such as CBT, the therapeutic alliance itself is less relevant to therapy outcome than factors related to the content of therapy. In the present study, we possibly did not find a development of therapeutic relationship over session number due to our highly structured and directive cognitive–behavioral program focusing mainly on psychoeducation, problem solving, and cognitive restructuring (55). Thus, the therapeutic relationship was less prominent than the motivational clarification and active assistance in problem solving at least in our short-term program in inpatient setting. As an alternative, development for the therapeutic relationship might not change over therapy sessions in the present study, because there is only little change in the empathy/alliance for each other, especially in our short-term treatment consisting of only eight sessions. The course of psychotherapy over the eight therapy sessions in 4 weeks seems therefore to be too short term to improve relationship between patient and therapist. Furthermore, among those few studies investigating direct changes of therapeutic alliance during the course of the therapy, it has been shown that therapeutic alliance might be rather stable (56).

Most importantly, evaluations of motivational clarification and active assistance in problem solving differed between patient groups with and without clinically relevant improvement of depressive symptoms. This indicates that the baseline levels in these variables were related to the treatment response (48). In contrast, the lack of a significant interaction between time and improvement of depressive symptoms indicate that the development of these common factors across time was not associated with the treatment response. However, we found marginally significant interactions between clinical improvement and session number for the scales active assistance in problem solving and motivational clarification. This interaction suggests that an increase in these factors over therapy sessions tended to be larger in the patient group with no clinically relevant improvement. These patients, who exhibited low baseline levels in the mentioned common factors, showed a stronger increase in those factors than the patients with clinically relevant improvement, who generally had higher scores on problem solving and motivational clarification. The analyses relating the slopes of the individual regression functions of the STEP scales to clinical improvement (BDI-II difference T0 − T1) failed to find significant associations. This indicates that the development of common factors over therapy sessions was not related to clinical improvement within this short-term therapy. It must remain open whether in a therapeutic treatment of longer duration; an increase of these common factors would be associated with an improvement of depressive symptoms.

When interpreting the results of our study, several limitations should be considered. First, the sample size (25 patients) was relatively small and did not allow testing the relation of improvements of depressive symptoms to common factors within patient subgroups. The small sample size also may give rise to a potentially high likelihood of false-negative results. Second, due to the lack of a control group or an active comparison group, the improvement in depressive symptoms and the change in common factors cannot be unequivocally referred to the psychotherapeutic treatment. The present research should therefore be considered best as a pilot study for the role of common factors in the short-term therapy of inpatients with major depression in a naturalistic inpatient setting. Therefore, future studies investigating this issue within a control group design and large sample size are clearly necessary to validate the current results. Third, psychotherapy sessions were conducted by PiT, which could have reduced the effectiveness of psychotherapy. Even though there is no evidence that psychotherapists with more experience achieve better therapy outcomes than psychotherapists with less experience (57), the involvement of PiT and many different PiT in our study could be a methodically important confounding factor. Fourth, the influence of pharmacological treatment within the study period on clinical improvement could not be quantified in our study. It might be possible that differential effectiveness of antidepressive medication increases interindividual variance in clinical improvement, which reduces the relevance of the development of common factors across therapeutic sessions. In future studies, the influence of antidepressant medication should be better controlled. Fifth, due to the small sample size of our pilot study, the influence of demographic patient characteristics such as gender or education and psychopathological characteristics such as number of depressive episodes could not be assessed. Future study should investigate whether we observe similar results in the group of homogeneous patients. Finally, it remains open whether the present findings generalize to other forms of psychotherapy as realized in the present study. In particular, we decided to assess development of common factors as well as depressive symptoms improvement during a short-term therapy over only 4 weeks because this treatment duration conforms to a typical stay of MDD patients in our hospital. However, a long-term follow-up assessment after completion of therapeutic sessions is needed in order to capture long-lasting effects. It must therefore remain open whether the common factors of psychotherapy unfold their effect on patients’ clinical improvement after a longer period of time. We are aware of the abovementioned limitations and potentially confounding factors in our pilot study, in particular the limited statistical power due to the small sample size. Thus, the present results need to be interpreted with care.

Taken together, the present pilot study showed that common factors can be measured in a short-term psychotherapy across eight sessions in a psychiatric inpatient setting. Furthermore, among the common factors, motivational clarification and active assistance in problem solving increased over therapy sessions, whereas therapeutic relationship did not show such a development during the study period of 4 weeks. Most importantly, baseline level of motivational clarification and active assistance in problem solving were positively related to the outcome of the therapy (i.e., the improvement in depressive symptoms). This highlights the distinguished role of these common factors for a positive treatment outcome similar to earlier work (58). Hence, our study indicates that these common factors are relevant for a successful treatment of depression and their consolidation should be explicitly part of psychotherapy. Nevertheless, it should be emphasized that the present work can only be considered as a pilot study due to the small sample size and lack of control for potentially confounding demographic or psychopathological variables. More research is therefore needed to investigate the influence of common factor on treatment outcome in a larger sample of inpatients with MDD.

## Author Contributions

KW, E-JS, and MK contributed to the study design. KW, E-JS, CS-L, and ZS-V contributed to data collection. KW, E-JS, FK, and MK conducted the data analyses. All authors contributed to interpretation of the data and drafting of the manuscript, and approved the final version.

## Conflict of Interest Statement

The authors declare that the research was conducted in the absence of any commercial or financial relationships that could be construed as a potential conflict of interest.
